# Characterization of Modified Mechanically Activated Cassava Starch Magnetic Porous Microspheres and Its Adsorption for Cd(II) Ions

**DOI:** 10.3390/nano13030513

**Published:** 2023-01-27

**Authors:** Xinling Xie, Xiaona Zhao, Xuan Luo, Youquan Zhang, Zuzeng Qin, Hongbing Ji

**Affiliations:** 1 Guangxi Key Laboratory of Petrochemical Resource Processing and Process Intensification Technology, School of Chemistry and Chemical Engineering, Guangxi University, Nanning 530004, China; 2 Fine Chemical Industry Research Institute, School of Chemistry, Sun Yat-sen University, Guangzhou 510275, China

**Keywords:** magnetic microspheres, cassava starch, inverse emulsion, polyethylene glycol/methanol system, adsorption

## Abstract

The magnetic polymer microsphere is a promising adsorbent due to its high adsorption efficiency and good regeneration ability from wastewater. Cassava starch magnetic porous microspheres (AAM-MSMPMs) were synthesized by graft copolymerization in inverse emulsion. Mechanically activated cassava starch (MS) was used to graft skeletons, vinyl monomers [acrylic acid (AA) and acrylamide (AM)] as copolymerized unsaturated monomers, methyl methacrylate (MMA) as the dispersing agent, and polyethylene glycol/methanol (PEG2000/MeOH) as the porogen. It was found that the AAM-MSMPM adsorbent is superparamagnetic, the saturation magnetization is 14.9 emu·g^–1^, and it can be rapidly and directionally separated from Cd(II) ions in aqueous solution. The FTIR indicated that the carboxyl and hydroxyl groups were grafted into MS. The AAM-MSMPM had good speroidization and a uniform size. After the porogen was added, the particle size of the AAM-MSMPM decreased from 19.00 to 7.00 nm, and the specific surface area increased from 7.00 to 35.00 m^2^·g^–1^. The pore volume increased from 0.03 to 0.13 cm^3^·g^–1^. The AAM-MSMPM exhibited a large specific surface area and provided more adsorption active sites for Cd(II) ions. The maximum adsorption capacity of the AAM-MSMPM for Cd(II) ions was 210.68 mg·g^–1^, i.e., 81.02% higher than that without porogen. Additionally, the Cd(II) ion adsorption process on the AAM-MSMPM can be described by Langmuir isothermal and pseudo-second-order kinetic models. A chemical reaction dominated the Cd(II) ion adsorption process on the AAM-MSMPM, and chemisorption was the rate-controlling step during the Cd(II) ion adsorption process. The AAM-MSMPM still had excellent stability after five consecutive reuses.

## 1. Introduction

The rapidly developed industry brought economic interest and caused severe environmental problems, especially the pollution of heavy metal ions [1,2,3]. Cadmium is one of the main environmental pollutants. Mineral processing wastewater containing cadmium and related industrial (electroplating alkaline battery) wastewater from lead–zinc mines alongside seriously polluting water and soil sources, among others [4,5]. Cadmium can be enriched in the organism, form cadmium thioprotein in the body, reach the whole body through the blood, and selectively accumulate in the kidney and liver, causing chronic poisoning [6,7]. Adsorption, complexation, photodegradation, electrodialysis, reverse osmosis, etc., have been used to treat heavy metal ion wastewater [8,9,10]. The adsorption method is routinely considered cost-effective and easy to operate, with temperate-operating conditions, high removal efficiency, and recyclability [11]. Polymer magnetic microspheres are a new type of adsorbent for heavy metals. Its chemical functional groups can provide more adsorption sites. Magnetic particles can be quickly and directionally separated from wastewater, making them a promising absorbent [12,13,14,15,16,17,18]. However, the current magnetic adsorbent has low adsorption efficiency and high cost due to its low surface area and other structural characteristics. Therefore, this study prepared a biobased magnetic porous adsorbent, which not only reduced the cost but also increased the specific surface area to further improve its adsorption capacity. Cassava starch is a natural polymer, and its products in nature are second only to cellulose. Because of its low cost, chemical modification renewability, and biodegradability, it has been widely used in industry [19,20,21]. However, the directional arrangements of crystal region molecules in starch molecules reduce the chemical reactivity of starch in industry due to its high gelatinization temperature, poor cold-water solubility, and low chemical reaction reactivity. Mechanically activated starch is a kind of physically modified starch. The crystalline structure of starch molecules is destroyed. The crystallinity decreases by the action of mechanical forces such as friction, collision, impact, and shearing, and the morphology of starch molecules is transformed from a polycrystalline state to an amorphous state. The size, shape, appearance, crystal structure, and molecular structure of starch granules treated by mechanical activation will change to varying degrees, making it easier for reaction reagent molecules to enter the interior of the chemical reaction [22,23,24]. Emulsion polymerization has the following advantages: mild reaction conditions, a stable reaction system, a rapid reaction rate, a high molecular weight of products, good spheroidization, and uniform particle size [23]. Therefore, this study used mechanically activated cassava starch as a raw material to prepare a new magnetic composite consisting of Fe_3_O_4_ magnetic particles, a graft copolymer of cassava starch, and vinyl monomers by inverse emulsion polymerization. It has the dual properties of polymers and magnetic particles and was used to adsorb Cd(II) ions in wastewater.

Therefore, inverse emulsion polymerization was used in this study to prepare a new magnetic starch microsphere adsorbent. The magnetic starch microsphere is a magnetic composite material composed of Fe_3_O_4_ magnetic particles, a graft copolymer of cassava starch, and vinyl monomers. Therefore, different studies have used the composite materials of polymers and magnetic particles to improve the adsorption capacity for heavy metal ions.

The specific surface area and particle size of adsorbents can improve the adsorption capacity and rate. It is still challenging to accurately construct multifunctional nanoparticles with small particle sizes, homogeneity, and uniform dispersion [25,26]. Nevertheless, the specific surface area, particle size, and pore volume of magnetic starch microspheres still need to be further improved. Adding the porogen to prepare a porous structure through chemical modification is promising. There are few reports on magnetic porous starch nanocomposite adsorbents.

In this work, a cassava starch magnetic porous microsphere adsorbent (AAM-MSMPM) was prepared by inverse emulsion crosslinking, with mechanically activated starch as the graft skeleton, polyethylene glycol (PEG) and MeOH binary system as the porogen, and acrylic acid (AA) and acrylamide (AM) as the grafted monomers. The adsorbent (AAM-MSMPM) was analyzed by Fourier transform infrared (FTIR) spectroscopy, scanning electron microscopy (SEM), X-ray photoelectron spectroscopy (XPS), and vibrating sample magnetometry (VSM). The adsorption isotherm and kinetic model were used to investigate the adsorption process and adsorption mechanism of Cd(II) ions on the AAM-MSMPM adsorbent.

## 2. Materials and Methods

### 2.1. Materials

Cassava starch was purchased from Shanghai Yuanye Technology Co., Ltd. (Shanghai, China). AM and liquid paraffin were obtained from Damao Chemical Reagent Co., Ltd. (Tianjin, China). Ammonium peroxydisulfate (APS), linear-alkylbenzenesulfonic acid (Op-4), AA, and PEG were obtained from Xilong Chemical Reagent Co., Ltd. (Shantou, China). Sodium hydroxide was purchased from Guanghua Technology Co., Ltd. (Guangdong, China). Acetone and methanol were obtained from Kelong Chemical Reagent Co., Ltd. (Chengdu, China). *N*, *N*’-Methylenebisacrylamide (MBAA) and sorbitan fatty acid ester (Span-80) were purchased from Macklin Reagent Co., Ltd. (Shanghai, China). All the chemicals were analytical grade without further purification throughout the study.

### 2.2. Synthesis of the AAM-MSMPM Absorbent

According to the literature, mechanically activated cassava starch (MS) was prepared [27]. The AAM-MSMPM was prepared using the procedure described in the literature [27] with minor modifications. A 1:3.5–1:2 (1 wt%) porogen (PEG/MeOH) agent was added to the reaction system as the porogen. The other preparation steps were the same as those in the literature [27].

### 2.3. Characterization of the AAM-MSMPM Absorbent

A vibrating sample magnetometer (VSM, 7410, LakeShore, Columbus, OH, USA) was used to measure the magnetic intensity of the AAM-MSMPM. Fourier transform infrared (FTIR) spectroscopy (Tensor II, Bruker, Billerica, Germany) was conducted in a scanning range of 4000 to 400 cm^–1^. The surface morphology of the adsorbents was measured at an accelerating voltage of 5.0 kV using scanning electron microscopy (SEM, SU-8220, Hitachi, Tokyo, Japan). The surface element composition and element valence of the AAM-MSMPM were measured in an Al *K*α ray source and C 1*s* (284.6 eV) for calibration, using a photoelectron spectrometer (XPS, ESCALAB 250Xi, Thermo Fisher Scientific Inc., Carlsbad, CA, USA). The N_2_–BET surface areas and pore size distributions of the adsorbent were determined by N_2_ adsorption–desorption on an ASAP 2460 physisorption analyzer (Micromeritics, Norcross, GA, USA), and the specific surface area of the AAM-MSMPM was calculated by the Brunauer–Emmett–Teller (BET) equation. The Barrett–Joyner–Halenda (BJH) equation was used to calculate the pore volume and size distribution.

### 2.4. Adsorption of the AAM-MSMPM for Cd(II) Ions

Adsorption experiments of the adsorbent for Cd(II) ions were carried out according to the literature [27].

The adsorption capacity of the AAM-MSMPM absorbent for Cd(II) ions was calculated by Equation (1).

(1) $$q_{t}=\frac{\left(c_{0}-c_{t}\right)\times V}{m}$$

where *q*_t_ is the adsorption capacity of the AAM-MSMPM for Cd(II) ions, mg·g^–1^; *c*_0_ and *c*_t_ are the initial concentrations of the Cd(II) ions and the concentration at adsorption time *t*, mg·L^–1^; *m* is the amount of AAM-MSMPM adsorbent, g; and *V* is the adsorbed solution volume, L.

## 3. Results and Discussion

### 3.1. Adsorbent Characterization

**Figure 1:**
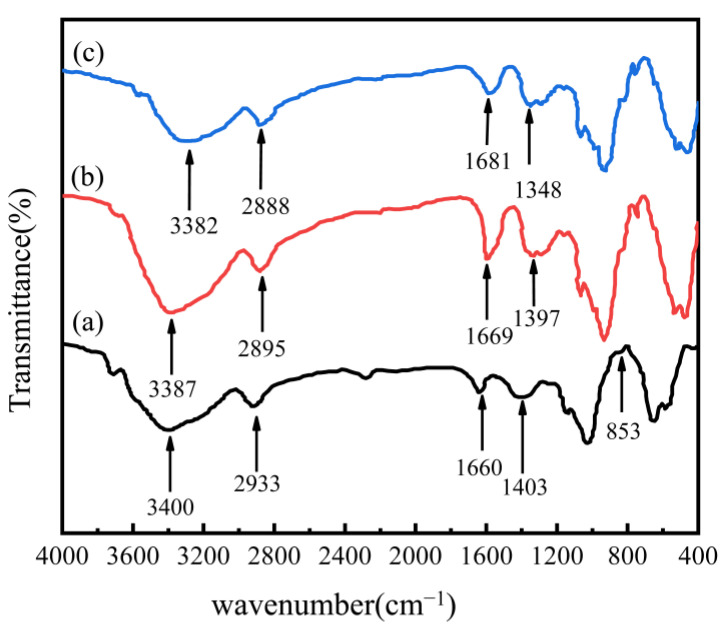
FTIR spectra for AAM-MSM (**a**), AAM-MSMPM (**b**), and Cd(II)/AAM-MSMPM (**c**).

The FTIR transmission spectra of AAM-MSM (without porogen) (a), AAM-MSMPM (b), and Cd(II)/AAM-MSMPM (AAM-MSMPM adsorbed with Cd(II), namely Cd(II)/AAM-MSMPM) (c) are shown in Figure 1. Figure 1a–c show that AAM-MSMs (a), AAM-MSMPMs (b), and Cd(II)/AAM-MSMPMs (c) all retain the characteristic adsorption peak of starch. In Figure 1a, 1660 cm^–1^ is the carbonyl peak and O-H bending vibration peak, showing a bimodal peak due to the mutual influence between the two peaks. Compared with Figure 1a, only the peak area at 1669 cm^–1^ of Figure 1b has a certain displacement. Other characteristic peaks did not change significantly, indicating that PEG/MeOH is physically porous and does not change the chemical structure of the adsorbent. In Figure 1c, the FTIR spectrum of the AAM-MSMPM adsorbed with Cd(II) ions (namely Cd/AAM-MSMPM) showed that the peak at 1669 cm^–1^ moved toward 1681 cm^–1^ in the direction of the high wave, because the adsorption of Cd(II) ions leads to an increase in the spatial hindrance of the peak vibration [28].

**Figure 2:**
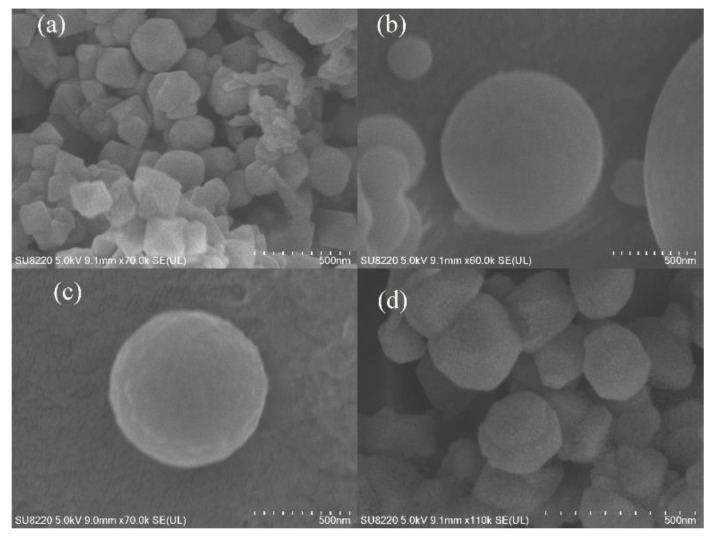
SEM images of AAM-MSMPM (PEG/MeOH = 15 wt%) (**a**), AAM-MSMPM (PEG/MeOH = 20 wt%) (**b**,**c**), and Cd(II)/AAM-MSMPM (**d**).

The SEM images of the AAM-MSMPM are shown in Figure 2. As shown in Figure 2, the amount of porogen significantly impacted the structure and morphology of the AAM-MSMPM. In Figure 2a, the porogen amount was 15 wt%, and the AAM-MSMPM formed balls, but some balls slightly adhered. In Figure 2b,c, the AAM-MSMPM showed good dispersion and pelletization when the amount of porogen was 20 wt%. The adsorption capacity of Cd(II) ions was also largest. Compared with AAM-MSM (without porogen), the pore diameter was reduced. However, the specific surface area and pore volume of the AAM-MSMPM were enhanced. The reason is that adding the porogen reduces the droplet size in the emulsion and alleviates the adhesion problem of starch magnetic microspheres caused by polymer and starch molecules [27]. Figure 2d shows that the surface morphology of the AAM-MSMPM that adsorbed Cd(II) ions presented a rougher surface morphology with some small bulged particles, indicating the successful adsorption of Cd(II) ions by the AAM-MSMPM. The finding has the same analysis results as XPS and FTIR.

**Figure 3:**
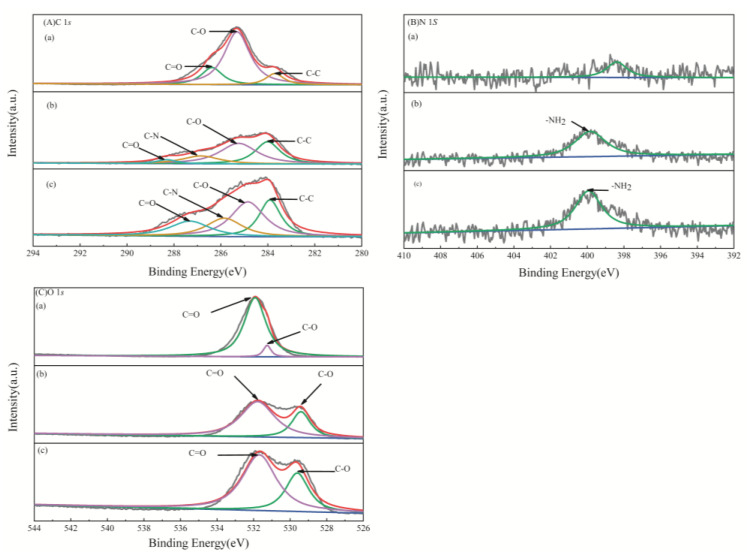
XPS profiles of C 1*s* (**A**), O 1*s* (**B**), and N 1*s* (**C**) in the MS (**a**), AAM-MSMPM (**b**), and Cd(II)/AAM-MSMPM (**c**).

To further elucidate the adsorption mechanism, the MS, AAM-MSMPM, and Cd(II)/AAM-MSMPM were characterized by XPS. The results are shown in Figure 3 and Table 1. It can be seen from Figure 3A that the C 1*s* spectrum of the MS comprised three fitting peaks, C–C, C–O, and C=O, centered at binding energies 283.73, 285.35, and 286.42 eV, respectively. The O1*s* spectrum of the MS had two fitting peaks, C–O and C=O, centered at 531.96 and 531.28 eV, respectively. There was no obvious peak in N 1*s*, indicating nearly no nitrogen in MS [29]. The XPS pattern of the AAM-MSMPM in Figure 3B exhibited a noticeable change, and the C–N signal peak also obviously appeared in the C 1*s* spectrum. The peaks, corresponding to C–C, C–O, C=O, and C–N, were fitted in the C 1*s* spectrum of the AAM-MSMPM, which were centered at 284.01, 285.25, 286.85, and 288.31 eV, respectively [30]. The peak value of C–N in C 1*s* suggested that the AM was successfully grafted to MS. The N 1*s* spectrum showed –NH_2_ peaks, corresponding to 399.83 eV. The N element mainly came from acrylamide. For the O 1*s* spectrum, there were two peaks centered at 529.41 and 531.78 eV, corresponding to O=C and O–C, respectively [6]. Compared with the MS spectrum, the chemical shifts of C=O and C–O caused by the chemical reaction between MS and oxygenated compounds were observed in the AAM-MSMPM spectrum. These results were confirmed by the FTIR results [30]. Figure 3C shows the XPS profile of the Cd(II)/AAM-MSMPM. The C 1*s* spectrum consisted of four fitting peaks, C–C, C–O, C–N, and C=O, centered at binding energies of 283.91, 284.85, 287.32, and 282.82 eV, respectively. O 1*s* comprises two fitting peaks, O=C and O–C, centered on 529.61 and 531.69 eV, respectively. The binding energy of the N 1*s* spectrum was 399.92 eV [29].

Compared with the changes in the binding energy of these elements in the adsorption after Cd(II) ion adsorption, chemical shifts of C and O were found, indicating that all elements participate in the adsorption process. After the AAM-MSMPM adsorbed the Cd(II) ions, the binding energy of C=O exhibited a blueshift of 4.03 eV, indicating that C had an obvious electron-receiving tendency [31]. The binding energy of N 1*s* had a redshift of 0.09 eV, meaning that N has a noticeable tendency to lose electrons or share lone pair electrons [32], possibly because the five electrons in the outer layer of the N atom bonded in pairs, and the rest of the lone pair electrons could not easily form complexes with Cd(II) ions. During the process, N and Cd(II) ions shared electrons, decreasing N’s electron density, and increasing binding energy. In addition, after the AAM-MSMPM adsorbed Cd(II) ions, the binding energies of O=C and O–C increased by 0.19 eV and 0.09 eV, respectively, indicating that O shares electrons with Cd(II) ions in the adsorption process to form a coordination complex [28].

**Figure 4:**
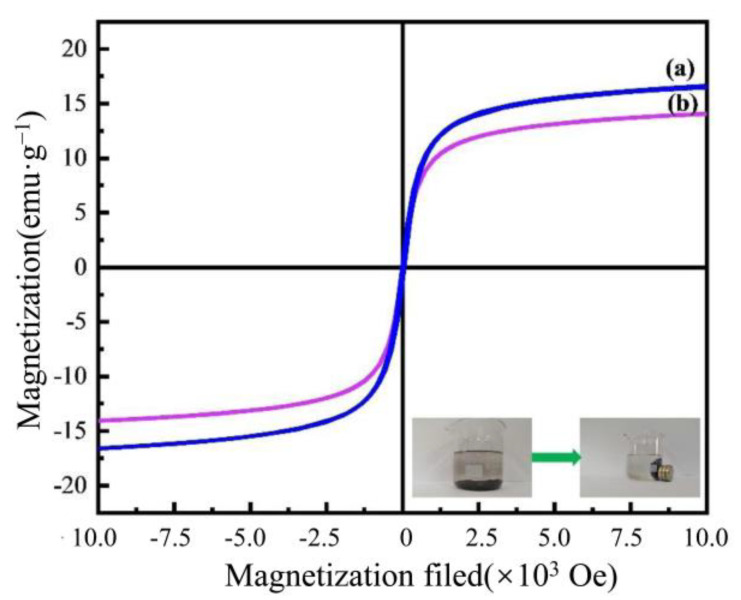
Magnetization curves of the AAM-MSMPM (**a**) and Cd(II)/AAM-MSMPM (**b**), and the inset of (**b**) is the separation of the AAM-MSMPM from the solution in a magnetic field.

Figure 4 shows the magnetic hysteresis curve of the AAM-MSMPM before and after adsorption. Figure 4 shows that the remanence and coercivity of the magnetization curves of the AAM-MSMPM before and after the adsorption of Cd(II) ions were zero. There is no hysteresis, indicating that the AAM-MSMPM is superparamagnetic before and after adsorption of Cd(II) ions [30]. For the AAM-MSMPM adsorption of Cd(II) ions, the saturation magnetization intensity was 16.9 emu·g^–1^. The magnet was attached to the outer wall of the beaker. The experimental results showed that all AAM-MSMPMs were attracted to the side of the magnet within 1 min, as shown in the inset of Figure 4. The Cd(II)/AAM-MSMPM saturation magnetization intensity was 14.9 emu·g^–1^. The magnetic hysteresis curve was similar to the AAM-MSMPM, indicating that the absorbent after adsorption for Cd(II) ions could still be readily separated and recycled from wastewater in a magnetic field.

**Figure 5:**
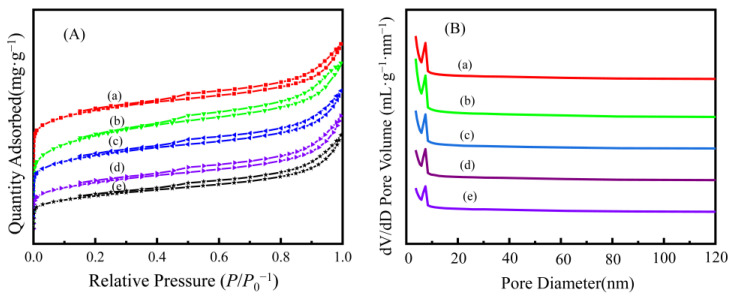
*N*_2_-adsorption–desorption isotherms (**A**) and pore distribution profiles (**B**) of the AAM-MSMPM (**a**, without porogen), (**b**, 10 wt% porogen), (**c**, 15 wt% porogen), (**d**, 20 wt% porogen), (**e**, 25 wt% porogen).

The specific surface area, pore size distribution, and pore volume were found to affect the adsorption capacity of the adsorbent [33]. Figure 5 shows the *N*_2_ adsorption–desorption isotherms and pore size distribution of the AAM-MSMPM. The specific surface area and pore analysis are shown in Table 2.

In Figure 5A, five *N*_2_ adsorption–desorption isotherm curves of the AAM-MSMPM were consistent with BEED Type-IV isothermal characteristics [34]. In the low-pressure stage of *P*/*P*^0^ = 0–0.4, there was a single-layer dispersed inflection point, while the middle area had a small slope. This region belonged to multilayer dispersion. The H4 hypospheric loop existed at *P*/*P*^0^ = 0.4–1.0, indicating that the AAM-MSMPM has a mesoporous structure. In Figure 5B, the pore diameters of the AAM-MSMPM were mainly distributed between 5 and 25 nm, with narrow pore size distribution, indicating that the AAM-MSMPM is a mesoporous material [35]. The introduction of PEG/MeOH changes the specific surface area and pore diameter, providing more active sites for the adsorption of Cd(II) ions. Table 2 shows that the specific surface area of the AAM-MSMPM increased from 7.00 m^2^·g^–1^ to 35.00 m^2^·g^–1^, the pore volume increased from 0.03 cm^3^·g^–1^ to 0.13 cm^3^·g^–1^, and the pore diameter decreased from 19.00 to 7.00 nm.

### 3.2. Effects of Adsorption Conditions

The surface of the AAM-MSMPM has active functional groups (–COOH, –OH, and –NH_2_), and the solution pH has a significant effect on its surface charge distribution and Cd(II) adsorption [36].

**Figure 6:**
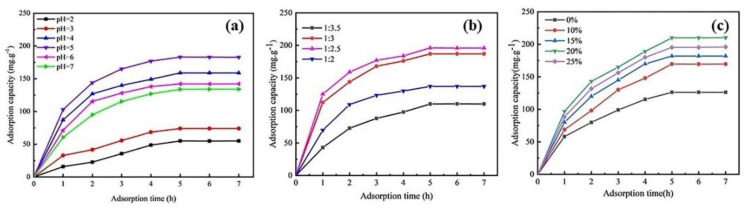
Effects of the solution pH (**a**), PEG/MeOH ratio (**b**), and the amount of monomer of PEG/MeOH (**c**) on the adsorption properties of Cd(II) on the AAM-MSMPM.

Figure 6a shows the effect of the solution pH within 2.0–7.0 on the Cd(II) ion adsorption capacity on the AAM-MSMPM. The Cd(II) ion adsorption capacity of the AAM-MSMPM increased from 54.42 mg·g^–1^ to 186.62 mg·g^–1^ as the pH of the solution increased from 2.0 to 5.0 and then decreased to 132.51 mg·g^–1^ as the pH of the solution increased from 5.0 to 7.0. The reason for this is the change in the surface chemical state of the adsorbent at different H^+^ ion concentrations. When the solution pH was less than 5, more H^+^ ions occupied the active adsorption sites, resulting in a loss of sites due to competitive adsorption with the Cd(II) cations in an acidic solution with a lower adsorption value. As the solution pH increased, the number of H^+^ ions decreased, which was conducive to the contact sites between the Cd(II) ions and the functional groups (–COOH, –OH, and –NH_2_) of the AAM-MSMPM surface, thus enhancing the adsorption capacity. However, when the pH was 5.0–7.0, the Cd(II) ions and the negative hydroxide ions formed hydroxide micro-precipitation formulations, reducing the adsorption capacity [37]. The maximum capacity of Cd(II) ions on AAM-MSMPM was 186.62 mg·g^–1^ at pH 5.0.

Figure 6b shows the effect of the mass ratio of PEG/MeOH on the AAM-MSMPM adsorption for Cd(II) ions. When the mass ratio of PEG to MeOH was reduced from 1:2 to 1:3.5, the adsorption capacity of the AAM-MSMPM for Cd(II) ions increased from 108.90 to 195.72 mg·g^–1^ and then decreased to 136.99 mg·g^–1^. When the mass ratio was 1:2.5, the maximum adsorption capacity for Cd(II) ions was 195.72 mg·g^–1^, as PEG is a macromolecular polymer. When the amount of MeOH decreased, the extensibility of PEG decreased. This exists in the solution as trackless wire group coils, resulting in decreased pore-forming capacity and adsorption capacity for Cd(II) ions [38]. When the mass ratio of PEG to MeOH continued to increase, the amount of MeOH was too high, the pore-forming ability of small molecule MeOH was limited, and the spheroidization was decreased, so the adsorption capacity of the AAM-MSMPM for Cd(II) ions decreased.

The amount of porogen had an important impact on the morphology and spheroidization of the adsorbent (as shown in Figure 2) and directly affected the adsorption efficiency. Figure 6c shows the effect of the porogen amount on the adsorption capacity of Cd(II) ions on the AAM-MSMPM. From Figure 6c, with increasing amounts of PEG/MeOH from 0 to 20 wt%, the Cd(II) ion adsorption capacity on the AAM-MSMPM rose from 125.77 to 210.68 mg·g^–1^, and then decreased to 182.26 mg·g^–1^. When the amount of PEG/MeOH was lower, the adsorption capacity of Cd(II) ions on the AAM-MSMPM was also low due to the small specific surface area of the AAM-MSMPM. When the amount of PEG/MeOH was 20 wt%, the chain relaxation of the porogen was expanded, which could play a better role in pore formation. The adsorption capacity was the highest, reaching 210.68 mg·g^–1^. When the amount of PEG/MeOH was higher than 20 wt%, the stability of the polymerization reaction system was reduced, so the pore-forming effect was weakened. The spheroidization of the adsorbent was reduced, so the adsorption sites on the surface of the AAM-MSMPM were reduced, resulting in a decrease in the adsorption capacity for Cd(II) ions [39].

### 3.3. Adsorption Isotherms of Cd(II) Ions on the AAM-MSMPM

The adsorption isotherm experiment was conducted at the initial Cd(II) ion concentrations from 10 to 90 mg·L^–1^ to investigate the adsorption behavior of the AAM-MSMPM adsorbent for Cd(II) ions. According to the effect of the initial Cd(II) ion concentration on the adsorption capacity, the adsorption process equilibrium data obtained by fitting were evaluated by the Langmuir equation and the Freundlich equation, respectively, as shown in Equations (2) and (3) [40].

(2) $$\frac{c_{e}}{q_{e}}=\frac{c_{e}}{q_{m}}+\frac{1}{K_{L}q_{m}}$$

(3) $$\log q_{e}=\log K_{F}+\frac{\log c_{e}}{n}$$

where *c*_e_ is the residual metal ion concentration at adsorption equilibrium, mg·L^–1^; *q*_e_ is the adsorption capacity at adsorption equilibrium, mg·g^–1^; *q*_m_ is the maximum adsorption amount, mg·g^–1^; *K*_L_ is the Langmuir adsorption equilibrium constant to characterize the adsorption capacity, L·mg^–1^; *K*_F_ is the Freundlich adsorption capacity constant, mg·g^–1^; and 1/n is the heterogeneity factor, which is related to the adsorption strength and the heterogeneity of the material surface.

**Figure 7:**
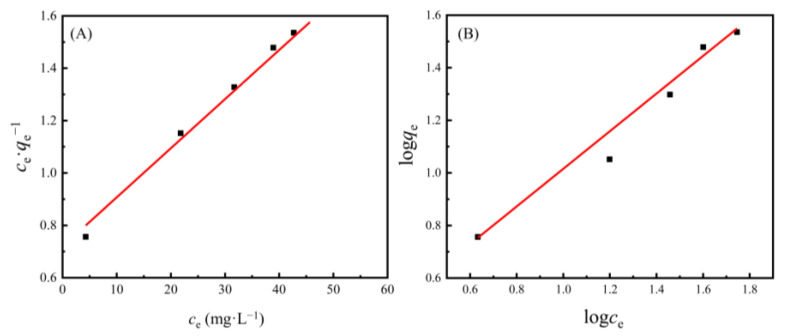
Fitting curve of (**A**) the Langmuir and (**B**) Freundlich isothermal adsorption models.

The correlation fitting results of the adsorption experimental data are shown in Figure 7. The correlation coefficients of the Langmuir and Freundlich models are shown in Table 3. In Table 3, the correlation coefficient *R*_2_^2^ obtained by Freundlich equation fitting was *R*_2_^2^ = 0.9782 (<0.99), and the correlation coefficient obtained by Langmuir equation fitting was *R*_1_^2^ = 0.9963 (>0.99). The experimental results show that the Langmuir model can better describe the adsorption behavior of the AAM-MSMPM than the Freundlich model (*R*_1_^2^ > *R*_2_^2^ > 0.99). The adsorption behavior of the AAM-MSMPM adsorbent for Cd(II) ions was described by monomolecular adsorption. The homogeneous functional groups on the adsorbent surface, including –COOH, –OH, and –NH_2_, supply more adsorption active sites in the adsorption process, and improve the adsorption capacity due to the coordination chelation between these functional groups and Cd(II) ions in solution through electrons. The maximum adsorption capacity *q*_m_ of the AAM-MSMPM for Cd(II) ions was 198.60 mg·g^–1^ (Table 3), close to the experimental result of 210.68 mg·g^–1^, indicating that they are very consistent. The larger *K*_L_ value of the AAM-MSMPM can be attributed to the decrease in particle size, the increase in the specific surface area, the decrease in the steric hindrance, and the increased adsorption capacity for Cd(II) ions.

### 3.4. Adsorption Kinetics of Cd(II) Ions on the AAM-MSMPM

Adsorption kinetic models are commonly adopted to study the effect of adsorption contact time on the adsorption capacity of adsorbents and the adsorption process. Different adsorption mechanisms are typically used, such as mass transfer, chemical reaction, and particle diffusion. Adsorption kinetic experiments Cd(II) ions on the AAM-MSMPM were carried out in our study. The pseudo-first-order kinetic model [using Equation (4)] and pseudo-second-order kinetic model [using Equation (5)] [41].

(4) $$\frac{1}{q_{t}}=\frac{k_{1}}{q_{e}\cdot t}+\frac{1}{q_{e}}$$

(5) $$\frac{t}{q_{t}}=\frac{k_{1}}{k_{2}q_{e}^{2}}+\frac{t}{q_{e}}$$

where *k*_1_ is the rate constant of the pseudo-first-order rate, min^–1^; *k*_2_ is the rate constant of pseudo-second-order, g·mg^–1^·min^–1^; *q*_e_ is the adsorption capacity of adsorption equilibrium, mg·g^–1^; and *q*_t_ is the adsorption capacity at time *t*, mg·g^–1^.

**Figure 8:**
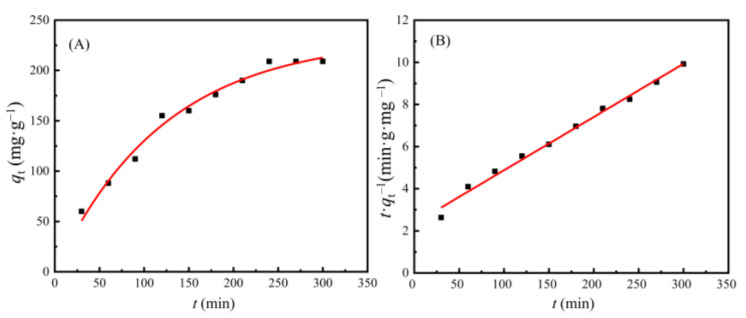
Adsorption kinetics of the AAM-MSMPM for the adsorption of Cd(II) ions: pseudo-first-order (**A**) and pseudo-second-order (**B**).

Figure 8 shows the adsorption kinetics of the AAM-MSMPM for Cd(II) ions. Figure 8A shows the plot between *q*_t_ and *t*. Using the slope and intercept of *t*/*q*_t_ and *t,* the pseudo-second-order rate constant (*k*_2_) and *q*_e_ can be calculated, as shown in Figure 8B. The relevant characteristic parameters of the fitting model and correlation are listed in Table 4.

Table 4 shows that the correlation coefficient *R*_1_^2^ obtained by the pseudo-first-order kinetic equation was 0.9826 (<0.99), demonstrating that the pseudo-first-order kinetic equation did not effectively describe the adsorption process. The correlation coefficient obtained by pseudo-second-order kinetic equation fitting was 0.9965 (>0.99), *R*_1_^2^ < *R*_2_^2^. Therefore, the pseudo-second-order kinetic equation was more suitable for describing the adsorption behavior of the AAM-MSMPM for Cd(II) ions than the pseudo-first-order kinetic equation.

According to Table 4, the adsorption capacity *q*_e_ was 232.61 mg·g^–1^ at equilibrium, which is close to the experimental data of 210.68 mg·g^–1^. It can be concluded that the adsorption of Cd(II) ions on the AAM-MSMPM was mainly due to chemical adsorption, which indicates that Cd(II) ions could be adsorbed on the AAM-MSMPM surface by functional groups –COOH, –OH, and –NH_2_. The XPS analysis also verified the results [42]. The adsorption capacity of Cd(II) ions on the AAM-MSMPM increased because of the addition of porogen.

### 3.5. Adsorption–Desorption Cycle Regeneration Performance of the AAM-MSMPM

**Figure 9:**
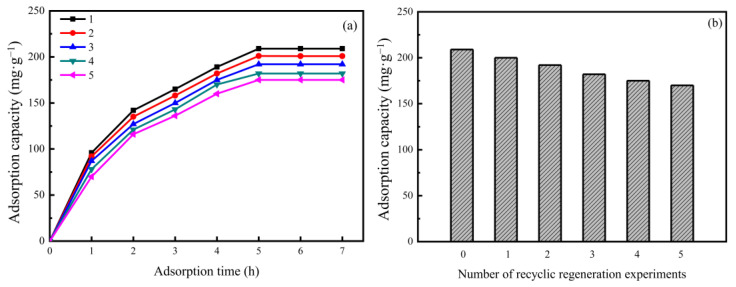
Desorption and regeneration experiments for the AAM-MSMPM: Effect of adsorption time at different recyclic numbers (**a**) and recyclic numbers (**b**) on the adsorption capacity.

The adsorption–desorption and recycling regeneration of the adsorbent is an important index to evaluate the adsorbent. After saturated adsorption, the adsorbent can be reused after desorption and regeneration, which can reduce the treatment cost and waste residue discharge and then recover the adsorbate, which is of great significance in actual industrial wastewater treatment [43]. Cd(II)/AAM-MSMPM adsorbents (0.1 g) were added to a 100 mL EDTA solution (0.1 mol·L^−1^) and placed on a constant temperature shaker for 12 h. The adsorption–desorption experiment was then repeated five times. Figure 9a shows the effect of the adsorption time on AAM-MSMPM adsorption for Cd(II) ions after the desorption–regeneration experiment. Figure 9b shows the reusability of AAM-MSMPM adsorption for Cd(II) ions in five desorption–regeneration cycles. The experimental results showed that after five recycling cycles, the AAM-MSMPM adsorption capacity for Cd(II) ions decreased from the original 210.68 to 170.56 mg·g^–1^, which maintained 80.95% of the original maximum adsorption capacity. Thus, the AAM-MSMPM has good reusability but cannot be completely desorbed because the loss of active sites of the AAM-MSMPM during desorption is the cause.

### 3.6. Comparison of the Adsorption Capacity of AAM-MSMPM with That of Other Adsorption Materials

In this study, the addition of the PEG/MeOH system reduced the particle size of the AAM-MSMPM. It increased the pore volume and specific surface area, resulting in a large Cd(II) ion adsorption capacity. The maximum adsorption of Cd(II) ions by different adsorbents is summarized in Table 5. The results showed that the adsorption capacity of the AAM-MSMPM for Cd(II) ions reached a maximum value of 210.68 mg·g^–1^ when Cd(II) adsorption on different adsorbents reached dynamic equilibrium. Compared with other adsorbents, it showed higher adsorption capacity. Because of its small particle size, large specific surface area, large pore volume, and certain adsorption functional groups, the AAM-MSMPM has the largest adsorption capacity and can be quickly separated and recovered from wastewater due to its magnetic properties. Therefore, the AAM-MSMPM is a promising adsorbent that can be used to purify heavy-metal-contaminated wastewater.

### 3.7. Adsorption Mechanism Analysis

**Figure 10:**
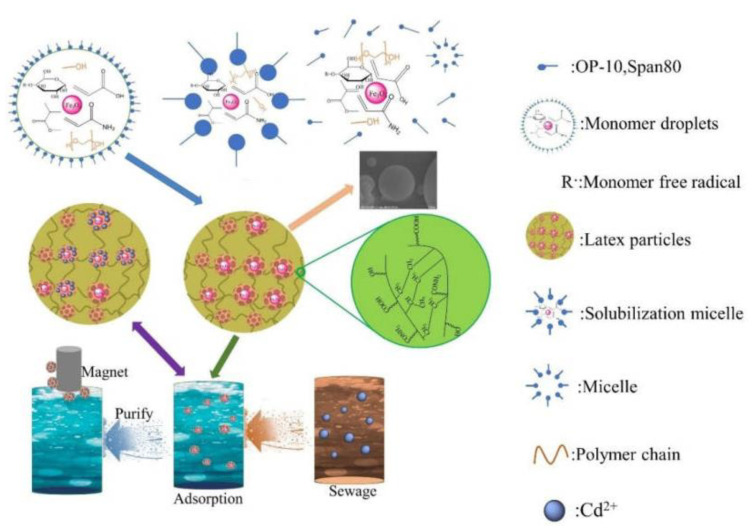
Schematic diagram of the AAM-MSMPM adsorption process.

The formation and adsorption mechanism of the AAM-MSMPM are shown in Figure 10. Based on the emulsion polymerization theory, the initiator APS initiated the starch and vinyl monomers (AM, AA, and MMA) to generate starch free radicals and monomer free radicals, respectively. Then, due to free radical chain polymerization, vinyl monomers were grafted into starch molecules, giving the AAM-MSMPM more functional groups (–NH_2_, –COOH, and –OH) and enhancing the adsorption of Cd(II) ions. The porogen agent was introduced into the copolymerization process. Then, the porogen agent was extracted, and finally, the AAM-MSMPM was prepared, as shown in Figure 10.

With the addition of porogen, the AAM-MSMPM surface provided more active adsorption sites and channels. The adsorption rate and adsorption capacity were increased, enhancing the adsorption of Cd(II) ions. In the initial stage of AAM-MSMPM adsorption, the adsorption process of Cd(II) ions experienced four steps: external diffusion, surface diffusion, pore diffusion, and adsorption. After Cd(II) ions diffuse to the adsorption sites on the surface, they are complexed with the functional groups on the surface of the AAM-MSMPM to form an adsorption reaction. When the concentration of the adsorbent in the solution and the concentration on the adsorbent surface no longer change, the adsorption process reaches dynamic equilibrium.

## 4. Conclusions

Magnetic cassava starch porous microspheres (AAM-MSMPMs) were prepared by polymerization in inverse emulsion, and the adsorption capacity for Cd(II) ions in simulated wastewater was studied. The AAM-MSMPM displayed a uniform size, smooth surface, regular shape, and good dispersion. The addition of the pore system (PEG/MeOH) gave the adsorbent a larger specific surface area and pore volume and a smaller pore diameter. The AAM-MSMPM exhibited a high Cd(II) ion adsorption capacity of 210.68 mg·g^–1^ and had good magnetic responsiveness and rapid separation from wastewater. The adsorption process of the AAM-MSMPM on Cd(II) ions was fitted well by Langmuir and pseudo-second-order models. The adsorption process of Cd(II) ions on the AAM-MSMPM was mainly chemical adsorption. In addition, the AAM-MSMPM had good reusability through five cycles of adsorption–desorption regeneration experiments, exhibiting an adsorption efficiency of 81% of the initial value.

## Figures and Tables

**Table 1:** Binding energies of the core electrons of the MS, AAM-MSMPM, and Cd(II)/AAM-MSMPM.

XPS Profiles	Element Valence	Binding Energy (eV)		
		MS	AAM-MSMPM	Cd(II)/AAM-MSMPM
C 1*s*	C–C	283.73	284.01	283.91
	C–O	285.35	285.25	284.85
	C–N	-	288.31	287.32
	C=O	286.42	286.85	282.82
N 1*s*	–NH_2_	-	399.83	399.92
O 1*s*	O=C	531.28	529.41	529.61
	O–C	531.96	531.78	531.69

**Table 2:** Texture properties of the AAM-MSM and AAM-MSMPM adsorbents.

Samples	BET Surface Area (m^2^·g^–1^)	Pore Volume (cm^3^·g^–1^)	Pore Size (nm)
AAM-MSM(a)	7.00	0.03	19.00
10 wt% AAM-MSMPM(b)	21.00	0.09	17.00
15 wt% AAM-MSMPM(c)	21.00	0.10	14.00
10 wt% AAM-MSMPM(d)	26.00	0.12	11.00
25 wt% AAM-MSMPM(e)	35.00	0.13	7.00

**Table 3:** Langmuir and Freundlich isothermal adsorption models for Cd(II) fitting parameters.

	*K*_L_ (L·mg^–1^)	*q*_m_ (mg·g^–1^)	*K*_F_	*n*	*R* ^2^
Langmuir model	0.0263	198.60	N/A	N/A	0.9963
Freundlich model	N/A	N/A	1.9951	1.3751	0.9782

**Table 4:** Adsorption dynamic model fitting parameters.

	*k*_1_ (min^–1^)	*q*_e_ (mg·g^–1^)	*k*_2_ (g·mg^–1^·min^–1^)	*q*_e_ (mg·g^–1^)	*R* ^2^
Pseudo-first-order	0.00891	232.6186	N/A	N/A	0.9826
Pseudo-second-order	N/A	N/A	2.7636 × 10^−4^	220.5011	0.9965

**Table 5:** Comparison of adsorption capacities for Cd(II) on the AAM-MSMPM with other adsorbents.

Different Kinds of Adsorbents	Adsorption Conditions			The Maximal Adsorption Capacity of Cd(II) (mg·g–1)	Ref.
	t (h)	pH	T (°C)		
Polyaniline grafted chitosan (PGC)	1.5	6	30	14.33	[43]
AAM-MSM	5	7	25	39.98	[27]
Fe_3_O_4_@PB	4	6	25 ± 1	9.25	[44]
Fe_3_O_4_/ATP	2	7	20	141.1	[45]
KMnO_4_-GL	5	6	25	48.82	[46]
AAM-MSMPM	5	5	55	210.68	This work

## Data Availability

The data presented in this study are available on request from the corresponding author.
